# Phenotypic, molecular, and in silico characterization of coumarin as carbapenemase inhibitor to fight carbapenem-resistant *Klebsiella pneumoniae*

**DOI:** 10.1186/s12866-024-03214-7

**Published:** 2024-02-27

**Authors:** Mahmoud Saad Abdel-Halim, Amira M. El-Ganiny, Basem Mansour, Galal Yahya, Hemat K. Abd El Latif, Momen Askoura

**Affiliations:** 1 Microbiology and Immunology Department, Faculty of Pharmacy, Zagazig University, Zagazig, 44519 Egypt; 2 Pharmaceutical Chemistry Department, Faculty of Pharmacy, Delta University for Science and Technology, Gamasa, 11152 Egypt

**Keywords:** *Klebsiella pneumoniae*, Carbapenem-resistant, Coumarin, qRT-PCR, Checkerboard assay, Molecular docking

## Abstract

**Background:**

Carbapenems represent the first line treatment of serious infections caused by drug-resistant *Klebsiella pneumoniae*. Carbapenem-resistant *K. pneumoniae* (CRKP) is one of the urgent threats to human health worldwide. The current study aims to evaluate the carbapenemase inhibitory potential of coumarin and to test its ability to restore meropenem activity against CRKP. Disk diffusion method was used to test the antimicrobial susceptibility of *K. pneumoniae* clinical isolates to various antibiotics. Carbapenemase genes (NDM-1, VIM-2, and OXA-9) were detected using PCR. The effect of sub-MIC of coumarin on CRKP isolates was performed using combined disk assay, enzyme inhibition assay, and checkerboard assay. In addition, qRT-PCR was used to estimate the coumarin effect on expression of carbapenemase genes. Molecular docking was used to confirm the interaction between coumarin and binding sites within three carbapenemases.

**Results:**

*K. pneumoniae* clinical isolates were found to be multi-drug resistant and showed high resistance to meropenem. All bacterial isolates harbor at least one carbapenemase-encoding gene. Coumarin significantly inhibited carbapenemases in the crude periplasmic extract of CRKP. The checkerboard assay indicated that coumarin-meropenem combination was synergistic exhibiting a fractional inhibitory concentration index ≤ 0.5. In addition, qRT-PCR results revealed that coumarin significantly decreased carbapenemase-genes expression. Molecular docking revealed that the binding energies of coumarin to NDM1, VIM-2, OXA-48 and OXA-9 showed a free binding energy of -7.8757, -7.1532, -6.2064 and − 7.4331 Kcal/mol, respectively.

**Conclusion:**

Coumarin rendered CRKP sensitive to meropenem as evidenced by its inhibitory action on hydrolytic activity and expression of carbapenemases. The current findings suggest that coumarin could be a possible solution to overcome carbapenems resistance in CRKP.

**Supplementary Information:**

The online version contains supplementary material available at 10.1186/s12866-024-03214-7.

## Introduction

Antimicrobial resistance (AMR) is an emerging worldwide crisis threatening human health [1]. By the year 2050, it is expected that infections caused by antimicrobial-resistant bacteria will result in approximately ten million deaths annually worldwide [2]. *Klebsiella pneumoniae* is an opportunistic, Gram negative bacteria that often causes various nosocomial infections [3]. *Klebsiella* can give rise to severe diseases in humans such as bloodstream infections (BSIs), liver abscesses, bacteremia, septicemia, meningitis, and soft tissue infections [4].

β-lactams are among the most prescribed antibiotics worldwide. Carbapenems are the most effective agents in β-lactams for treatment of infections caused by multiple drug-resistant (MDR) bacteria [5]. Carbapenems have a broader spectrum of antimicrobial activity than other β-lactam antibiotics and are the most effective against both Gram positive and Gram negative bacteria including *K. pneumoniae* [5]. As a result of their efficacy and safety, they are extensively used worldwide which has led to the increased emergence of microbial resistance to carbapenems representing a huge global public health issue [6].

Carbapenem resistant *Klebsiella pneumoniae* (CRKP) was listed at the top of the urgent threat pathogens in the center for disease control and prevention (CDC) antibiotic resistance (AR) report [7]. In addition, many reports demonstrated that CRKP is considered a serious threat to global health [8, 9]. Due to the lack of effective treatments for carbapenem resistant *Enterobacteriaceae* (CRE) infections, the mortality rates could be as high as 40 to 50% which also affects the financial costs of patients’ hospitalization [10].

Carbapenem resistance in Gram-negative bacteria can be acquired through various mechanisms. The first mechanism is to limit intracellular drug concentration by either reducing penetration through outer membrane [11], or antibiotic efflux [12]. The second one is to modify the intracellular antibiotic target through genetic mutations or post-translational modification of drug targets [13]. The production of carbapenemases which inactivate carbapenems is the third and common mechanism of bacterial resistance to carbapenems [14].

Carbapenemases are classified according to their molecular structure and amino acid sequences into three classes. Class A and D carbapenemases depend on the serine amino acid on the active pocket of the enzyme for carbapenem hydrolysis. Class B metallo *β*-lactamases (MBLs) such as New Delhi metallo *β*-lactamase (NDM) depend on divalent zinc ions for carbapenems hydrolysis [14]. Metallo *β*-lactamases (Class B) and oxacillinases (Class D) are considered the major contributors to carbapenem resistance [15]. In Egypt the most common carbapenemases are *bla*_*OXA*_ followed by *bla*_*VIM*_ and *bla*_*NDM*_ [15, 16]. The best way to overcome carbapenem resistance is to use combinations of carbapenem and *β*-lactamase inhibitors (BLI). Avibactam, relebactam, and vaborbactam are new clinically approved carbapenemase inhibitors with activity against CRE. The recently approved inhibitors are effective against several serine based enzymes; however, they are not effective against MBLs [17]. Therefore, there is an urgent need to search for novel MBLs inhibitors.

Compounds from natural sources were reported as BLIs in various studies [18–20]. One of the natural compounds that could exhibit a promising antibacterial application is coumarin. Coumarins occur naturally in cinnamon, tonka beans, sweet clover, and cassia cinnamon and were isolated for the first time as natural products in 1820 [21, 22]. Coumarins are aromatic compounds composed of fused heterocycles. One of these rings is benzene and the other comprises an alkene functionality and cyclic ester (δ lactone) which has been extensively reported as 2 H-chromen-2-one [23]. Coumarin and its derivatives exhibit various biological activities such as antibacterial [24, 25], antifungal [26, 27], anti-inflammatory as well as anticoagulant activity [28]. Coumarin is widely used as an additive in cosmetics, food flavor and perfumes [29]. Recently, the role of coumarins as quorum sensing inhibitors in several pathogens has been reported [30]. The present study aims to evaluate the inhibitory potential of coumarin against both class B MBLs (*bla*_*VIM*_ and *bla*_*NDM*_) and class D carbapenemase (*bla*_*OXA*_) and to characterize coumarin ability to restore meropenem activity against carbapenem resistant *K. pneumoniae*

## Materials and methods

### Bacterial isolates and chemicals

Six clinical *K. pneumoniae* isolates were included in the current study, these isolates were obtained from the culture collection of the Microbiology and Immunology Department, Faculty of Pharmacy, Zagazig University. These isolates were previously characterized using 16 S rRNA sequencing [18] and their sequences were submitted to GenBank (https://www.ncbi.nlm.nih.gov/) and given the following accession numbers: ON798797 (1 K), ON798798 (2 K), ON798799 (3 K), ON798800 (4 K), ON798801 (5 K), and ON798802 (6 K). Coumarin, dimethyl sulphoxide (DMSO), and resazurin were bought from Sigma Chemical Co. (St. Louis, MO, USA).

### Antibiotic susceptibility testing

The disk diffusion method was used to test the antibiotic sensitivity of *K. pneumonia*e isolates based on the clinical and laboratory standard institute (CLSI) guidelines [31]. Briefly, a sterile swab emerged in 0.5 McFarland standards equivalent of bacterial culture in Mueller Hinton (MH) broth was used to inoculate the surface of a MH agar plate and let to dry. The antibiotic disks; MEM: meropenem, TZP: piperacillin-tazobactam, CRO: ceftriaxone, FEP: cefepime, CFP: cefoperazone, ATM: aztreonam, GN: gentamicin, AK: amikacin, AZM: azithromycin, TE: tetracycline, TGC: tigecycline, LEV: levofloxacin, OFX: ofloxacin, SXT: trimethoprim-sulfamethoxazole, and C: chloramphenicol were purchased from (Oxoid, UK). Antibiotic disks were placed on the surface of the bacteria inoculated MH agar plates. Plates were incubated for 18 h at 37 °C and the diameters of growth inhibition zones were determined and interpreted according to CLSI [31].

### Carbapenemase detection using PCR

Three carbapenems resistant genes (*bla*_NDM_, *bla*_VIM_ and *bla*_OXA−9_) were tested in *K. pneumoniae* isolates using PCR. The primers used in this study (Table 1) were produced by IDT (Integrated DNA Technologies, Coralville, Iowa, USA). The PCR reaction mixture included 25 µL of the COSMO PCR RED 2x Master Mix (Willowfort, UK), 2µL of the forward and reverse primer, 2 µL of bacterial gDNA, and up to 50 µL nuclease-free water. The amplification cycle was set to 3 min at 95 °C as the initial denaturation temperature followed by repeated 30 cycles of: denaturation for 5 s at 95 °C, annealing at different temperatures (according to each primer as listed in Table 1) for 30 s as indicated in Table 1, and extension at 72 °C for 1 min, and a final 5 min of extension at 72 °C.

**Table 1:** Primers used in this study

Gene	Primer sequences (5’→3’)	Annealing temp.	Reference
16 S rRNA	F: ATCTTCGGACCTCACGCTATC R: TCATCCTCTCAGACCAGTTAC	50°C	[32]
*bla*_NDM_	F: GCACACTTCCTATCTCGACATGC R: CCATACCGCCCATCTTGTCC	51.5°C	[33]
*bla*_VIM_	F: GATGGTGTTTGGTCGCATA R: CGAATGCGCAGCACCAG	56°C	[34]
*bla*_OXA−9_	F: CGTCGCTCACCATATCTCCC R: CCTCTCGTGCTTTAGACCCG	51°C	[33]

### Determination of minimum inhibitory concentration (MIC) of meropenem and coumarin against *K. pneumoniae* isolates

The MIC of both meropenem (MEM) and coumarin against *K. pneumoniae* was determined by the broth micro-dilution method [31]. An overnight bacterial culture of *K. pneumoniae* in MH broth was diluted using sterile phosphate buffer saline (PBS) to 0.5 McFarland standards equivalent turbidity. Then 1:100 dilution (in sterile MH broth) of the bacterial suspension was prepared. Serial dilutions of meropenem (0.5–1024 µg/mL) and coumarin (16-8192 µg/mL) in sterile MH broth were prepared in sterile 96 wells microplates and 50 µL of freshly prepared bacterial suspension was introduced into each well. After incubation for 18 h at 37 °C, the results were recorded. The MIC of *K. pneumoniae* was determined as the lowest concentration of tested agent to inhibit visible bacterial growth [31].

### Characterization of the effect of coumarin sub-MIC on *K. pneumoniae* metabolic activity using the alamar blue assay

Alamar blue (resazurin) assay was used to evaluate the action of sub MIC of coumarin on the viability of *K. pneumoniae* isolates [35]. Bacterial culture was incubated with and without 500 and 1000 µg/mL of coumarin at 37 °C for 18 h followed by centrifugation at 8,000 rpm for 10 min. Cell pellets were collected and dispensed in freshly made PBS. A final volume of 100 µL resazurin stock solution prepared in PBS (6.5 mg/mL) was added to 900 µL of bacterial suspension. Finally, the mixture was incubated away of light at 37 °C for 4 h. As a control, resazurin in PBS was included without bacterial culture. The fluorescence intensity of reduced resazurin was measured using a microplate reader (synergy HT BioTek, Santa Clara, California, USA) at 590 nm emission and 560 nm excitation wavelengths.

### Combined disk test

This test was performed to assess the synergy between both meropenem and coumarin [36]. Briefly, MH agar plates containing various concentrations of coumarin (500 and 1000 µg/mL) were prepared and allowed to dry. The overnight bacterial culture in MH broth was diluted in PBS to a turbidity equivalent to that of 0.5 McFarland’s standard and was used to inoculate the surface of the MH agar plates using a sterile cotton swab. Finally, a disk containing 10 mg of meropenem was placed on the center of the plate and plates were incubated for 18 h at 37 °C. The diameters of bacterial growth inhibition zones were measured and photographed relative to plates with no coumarin.

### Carbapenemase inhibition assay of crude periplasmic extract in presence of coumarin

Crude periplasmic extracts of *K. pneumoniae* isolates were prepared as described before [37]. Briefly, overnight bacterial culture were centrifuged at 10,000 rpm for 10 min to harvest the cells. Bacterial cells were resuspended in 500 µL of phosphate buffer (100 mM, pH 7.0) with 50 µM ZnSO_4_ and sonicated (Ultrasonic System UP100H, Hielscher–Ultrasound Technology, Teltow, Germany) at 40 W, with a pulse of 0.5 s, during 1.5 min. Sonicated bacterial suspensions were centrifuged at 10,000 rpm for 10 min at 4 °C. The supernatants (100 µL) were then used to estimate the enzymes’ hydrolytic activity of meropenem upon incubation in a 96 well microtitre plate for 30 min with and without coumarin (1000 µg/mL) in 0.5% DMSO [38]. Then, 500 µg/mL of meropenem was added, the plate was set at 37 °C for 1 h in the incubator. The optical densities (OD) of solutions containing coumarin (treated) and 0.5% DMSO (control) were measured spectrophotometrically at 297 nm using UV-Vis spectrophotometer (synergy HT BioTek). The inhibition percentage of meropenem hydrolysis was calculated using the following formula: % of inhibition = [(OD of treated - OD of control)] x 100/OD of treated [19].

### Carbapenemase inhibition assay of crude periplasmic extract following co-culture of *K. pneumoniae* isolates with coumarin

Three tubes containing 10 mL MH broth were inoculated with overnight culture of *K. pneumoniae.* One tube contained 500 µg/mL of coumarin, the second tube contained 1000 µg/mL of coumarin and the third tube contained no coumarin as a control. Tubes were incubated at 37 °C with shaking for 24 h and centrifuged at 10,000 rpm for 10 min. Cell pellet was rinsed twice with phosphate buffer (100 mM, pH 7) with 50 µM ZnSO_4_ to remove any traces of coumarin followed by centrifugation to collect bacterial cells [37, 39]. Bacterial pellets were suspended in 100 mM phosphate buffer (pH 7.0) with 50 µM ZnSO_4_ and bacterial suspension was adjusted to a final count of 5 × 10^8^ CFU/mL [37] in a microfuge tube and sonicated at 40 W for 1 min, with a pulse of 2 s to disrupt cells and release the periplasmic enzymes [37].

Bacterial suspensions were then centrifuged at 10,000 rpm for 10 min at 4 °C and supernatant was collected for determination of meropenem hydrolysis activity [38]. Briefly, supernatant was added to wells in 96 well microtiter plate containing meropenem (500 µg/mL) [19]. Ethylene-diamine-tetra-acetic acid (EDTA) was added as an inhibitor of MBLs to separate the action of MBL from that of serine based class A and D carbapenemases. The experiment design was shown in supplementary Fig. 1. The reaction plate was incubated for 45 min [40], and the OD of solution was measured using spectrophotometer at 297 nm. The inhibition and hydrolysis percentage of meropenem were estimated as described previously using the following formulae: % of hydrolysis = 100-(% of inhibition) where % of inhibition = [(OD of treated - OD control)/OD of treated] x 100 [19].

### Checkerboard assay

Two-fold checkerboard microdilution method was performed for quantitative assay of the synergy between meropenem and coumarin following the method described in CLSI [31]. Briefly, two-fold serial dilutions of coumarin (final concentrations range from 0 to 4096 µg /mL in 100 µL of MH broth) were added to each column, and two-fold dilutions of meropenem (final concentrations range from 0 to 512 µg /mL in 100 µL of MH broth) were added in each row in a 96-well microtiter plate. Then, 100 µL of bacterial cells (1 × 10^6^ CFU/mL) were added to each well and incubated at 37 °C for 24 h. The MICs of both coumarin and meropenem were detected by measuring the turbidity of each well at 600 nm using a microplate reader. The fractional inhibitory concentration indices (FICI) were determined by the formula: FICI = (MIC of coumarin used in combination/MIC of coumarin used alone) + (MIC of meropenem used in combination/MIC of meropenem used alone).

### RNA isolation and quantitative real time PCR (qRT-PCR)

The qRT-PCR was run to estimate the effect of coumarin on the expression of cabapenemase genes (*bla*_NDM_, *bla*_VIM_ and *bla*_OXA− 9_). Briefly, 5 mL Luria-Bertani (LB) broth was inoculated with 0.5 mL of 0.5 McFarland-equivalent bacterial suspension and incubated at 37 ºC for 18 h to harvest cells. Total RNA was extracted using GeneJET RNA purification Kit (Thermo Fisher Scientific Inc. Germany), following the manufacturer’s instructions. Total RNA (2 µg) was used to generate cDNA by reverse transcription using random hexamer primers (ThermoFisher scientific, USA) in a final reaction volume of 20 µL according to manufacture guide of SuperScript™ II RT (Invitrogen™, California, USA). Quantified estimation of gene transcripts was conducted using the PowerUp™ SYBR™ Green Master Mix. (Applied Biosystems, Thermo Fisher Scientific) in Agilent Stratagene Mx3005P qPCR Cycler System 5 Color 96-Well (Santa Clara, California, USA). The primers used for qRT-PCR are indicated in Table 1. Fold changes in the expression of the tested genes were determined using the comparative Ct (2^*−ΔΔCt*^) method with 16 S rRNA employed as a housekeeping gene [41].

### Molecular docking study

The crystal structures of *Klebsiella pneumoniae* proteins; apo-NDM-1 at a resolution of 2.10 Å and Beta-lactamase VIM-2 in complex with (2R)-1-(2-Benzyl-3-mercaptopropanoyl) piperidine-2-carboxylic acid at a resolution of 1.50 Å were retrieved from the Protein Data Bank (PDB ID: 3SPU) and (PDB ID: 5O7N), respectively [42, 43]. In addition, the predicted Class D OXA-48 carbapenemase protein encoded by blaOXA gene of *Klebsiella pneumoniae* ( https://www.uniprot.org/uniprotkb/A0A809EUY3/entry) and the inferred from homology, Beta-lactamase protein, encoded by blaOXA-9 gene of *Klebsiella pneumoniae* (https://www.uniprot.org/uniprotkb/M9V370/entry) were obtained as 3D structure in PDB format from AlphaFold Protein Structure Database (https://alphafold.ebi.ac.uk/entry/A0A809EUY3), (https://alphafold.ebi.ac.uk/entry/M9V370) respectively.

For Ligand preparation, Coumarin and Meropenem were drawn into Marvin Sketch of Marvin suite (http://www.chemaxon.com) to generate the lowest energy conformer. Dock module of MOE (Molecular Operating Environment) version MOE 2019.0102,2 [44] was utilized in docking studies. All water molecules were removed from the target protein receptors, then 3D protonation was applied with their standard geometry to incorporate hydrogen atoms into the protein structure, followed by energy minimization as program’s default parameters [45, 46]. Both ligands were docked against the rigid binding pocket of the protein using flexible ligand mode. Poses from ligand conformations were generated from the placement phase [47] The free energy of binding of the ligand from a certain pose is estimated using the force field-based scoring function GBVI/WSA ΔG [48].

### Statistical analysis

GraphPad Prism version 5.0.1 for Windows, (GraphPad Software, San Diego, California USA) was used in the statistical analysis. The results of carbapenemase inhibition assay, alamar blue assay, and combined disk test were analyzed using one-way ANOVA followed by Dunnett’s post hoc test. RT-qPCR results were analyzed by two-way ANOVA with a *P* value < 0.05 was considered significant.

## Results

### Susceptibility testing of *K. pneumoniae* isolates to various antimicrobial agents

The disk diffusion method was performed on six *k. pneumoniae* isolates to determine the sensitivity of *K. pneumoniae* clinical isolates against different antibiotics. All *K. pneumoniae* isolates were found to be multi drug resistant (MDR) as these isolates were resistant to antibiotics belonging to 3 or more different antibiotic classes. Furthermore, the antibiotic susceptibility testing shows that all *K. pneumoniae* isolates tested herein were resistant to meropenem (Supplementary Table 1).

### Detection of carbapenemase encoding genes

Three carbapenemase-encoding genes were tested; two MBL-enzymes (*bla*_*NDM−1*_ & *bla*_*VIM−2*_) and one from class D (*bla*_*OXA−9*_). It was found that all *K. pneumoniae* isolates included in current study (1 to 6 K) harbor at least one carbapenemase-encoding gene. Three of these isolates (**1 K**, **5** and **6 K**) were found to harbor three carbapenemase genes. Two isolates (3 and 4 K) encode two genes; *bla*_*NDM−1*_ and *bla*_*VIM−2*_, while one isolate; **2 K** encodes only *bla*_*NDM−1*_ (Table 2).

**Table 2:** Detection of carbapenemase encoding genes by PCR

Isolate’s code	bla_OXA−9_	bla_NDM−1_	bla_VIM−2_
1 K	+	+	+
2 K	-	+	-
3 K	-	+	+
4 K	-	+	+
5 K	+	+	+
6 K	+	+	+

### Determination of minimum inhibitory concentration (MIC) of meropenem and coumarin against ***K. pneumoniae*** isolates

The MICs of meropenem (MEM) against *K. pneumoniae* isolates were identified by the broth micro-dilution method. The observed MICs of meropenem (MEM) ranged from 64 to 512 µg/mL. On the other hand, the MICs of coumarin against tested *K. pneumoniae* isolates were ≥ 8192 as shown in Table 3.

**Table 3:** Minimum inhibitory concentrations (MICs) of meropenem alone and in combination with coumarin and FIC index

Isolate’s code	Coumarin MIC (µg/mL)	MEM MIC (µg/mL)	MEM + coumarin		FIC index	
			500 µg/mL	1000 µg/mL	500 µg/mL	1000 µg/mL
1 K	≥ 8192	128	64	1	0.56	0.13
2 K		256	256	0.5	1.06	0.12
3 K		128	32	0.25	0.375	0.12
4 K		512	256	64	0.56	0.13
5 K		64	1	< 0.25	0.07	0.12
6 K		64	2	< 0.25	0.09	0.12

MEM: Meropenem; FIC: Fractional Inhibitory Concentration

### Effect of sub MIC of coumarin on *K. pneumoniae* cell viability with alamar blue assay

**Fig. 1:**
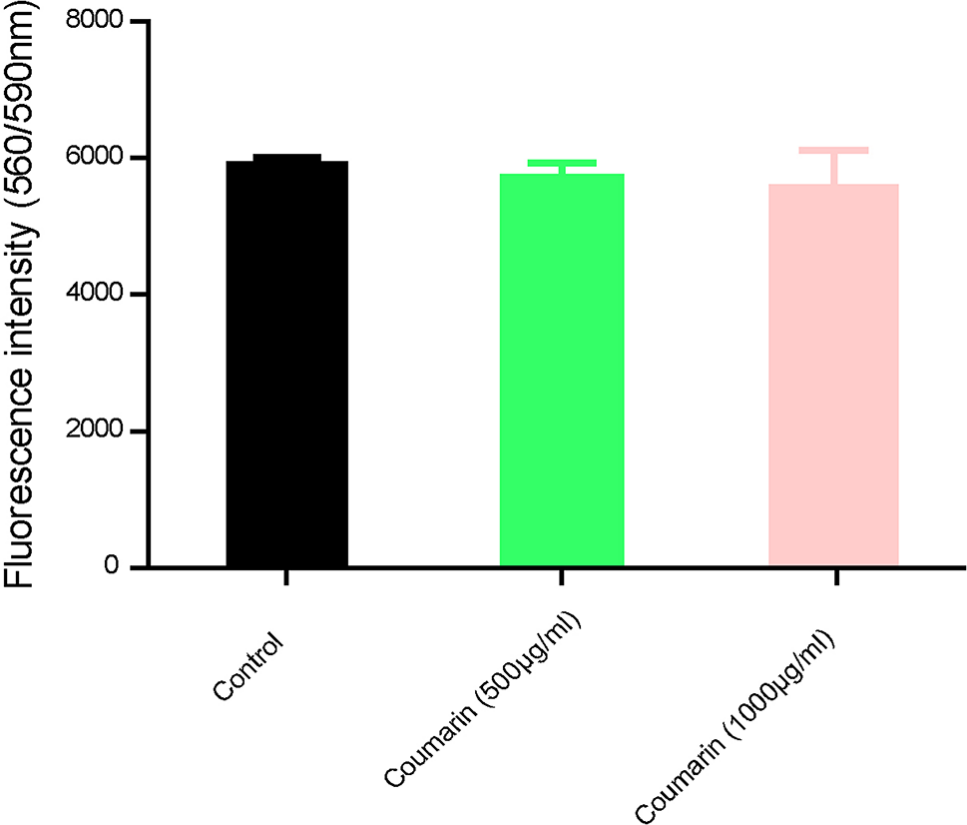
Alamar blue assay for bacterial viability assessment. There was no change in the fluorescence intensity between both coumarin-treated and untreated bacteria

Coumarin at sub-MICs of 500, 1000 µg/mL did not affect the viability of *K. pneumoniae* isolates. These findings were represented as a comparison of the fluorescence intensity of the reduced resazurin between coumarin-treated and untreated bacterial cultures as shown in Fig. 1.

### Coumarin inhibited carbapenemases activity in *K. pneumoniae* crude periplasmic extract

**Fig. 2:**
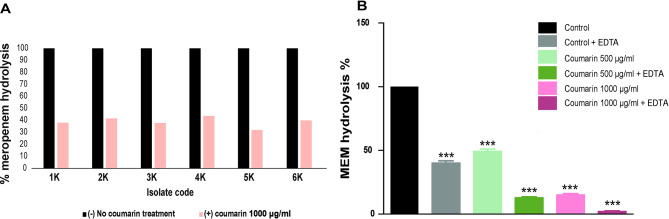
Carbapenemase enzyme inhibition assay **(A)** Comparison of percentages of meropenem hydrolysis in coumarin treated and untreated supernatants (co-incubation) **(B)** Comparison of percentages of meropenem hydrolysis in the supernatant of *K. Pneumoniae* grown in the presence and the absence of 500 or 1000 µg/mL coumarin (co-culture). EDTA was added to inhibit MBLs and to differentiate the action of MBL from that of serine-based class A and D carbapenemases. *** indicates *P* < 0.001

Coumarin (1000 µg/mL) markedly inhibited the hydrolytic potential of carbapenemases on meropenem in *K. pneumoniae* isolates crude periplasmic extract. The inhibition percentage among coumarin-treated isolates was (57.33% ± 7.59) as shown in Fig. 2A. In addition, *K. pneumoniae* isolates were co-cultured with sub-MIC of coumarin, then the bacterial periplasmic extract was collected and tested for meropenem hydrolysis activity. It was found that *K. pneumoniae* cells treated with coumarin exhibited a reduced meropenem hydrolysis (*P* < 0.001) as compared to the untreated bacteria (Fig. 2B).

### Coumarin synergizes meropenem activity agains *K. pneumoniae* isolates

**Fig. 3:**
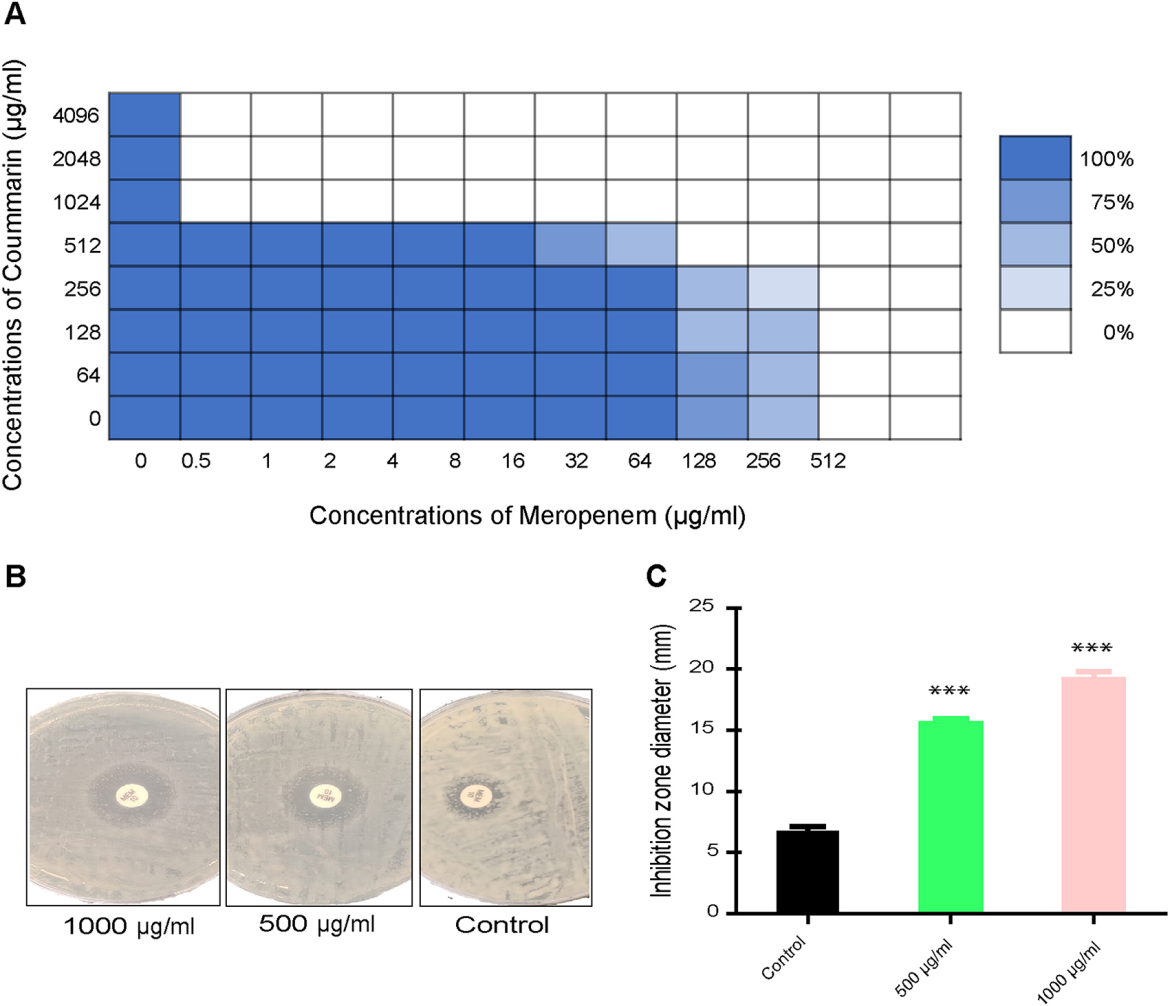
Effect of coumarin-meropenem combination on *K. pneumonia* isolates. **(A)** Checkerboard assay indicated a synergistic effect of coumarin-meropenem combination against carbapenemase positive *K. pneumoniae* isolates. The Fig. shows multiple possible combinations. The color gradient reflects the turbidity of bacterial culture (OD = 600 nm) **(B)** Inhibition zones surrounding meropenem disks on MH agar plates without and with two concentrations of coumarin (500 and 1000 µg/mL). **(C)** Coumarin significantly increased the diameter of bacterial growth inhibition zones surrounding meropenem disks relative to that of control without coumarin treatment. ***indicates *P* < 0.001

The combined disk test showed that the inhibition zone around meropenem disk increased significantly in a dose dependent manner when combined with coumarin (500 and 1000 µg/mL) as shown in Fig. 3 (B & C). Furthermore, in order to confirm the synergy between coumarin and meropenem, the checkerboard MIC assay was performed (Fig. 3A). The checkerboard assay results showed that coumarin at concentrations ≥ 1000 µg/mL resulted in the highest MIC fold change ranging from 8 to 256-fold decrease compared to the MIC of meropenem alone. The FICI values of this combination were 0.12–0.13 (< 0.5) for all tested *K. pneumoniae* isolates indicating a synergism as shown in Table 3.

### Coumarin significantly decreased the expression of carbapenemase genes

**Fig. 4:**
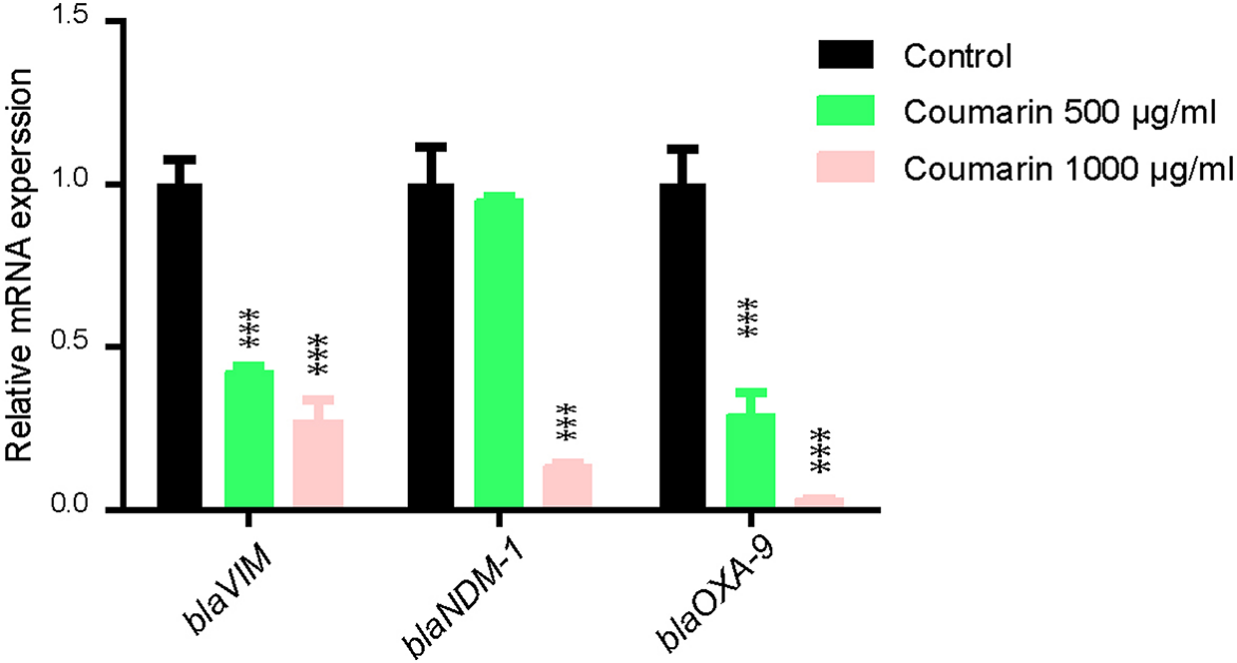
Coumarin decreased the expression of carbapenemase genes as revealed by qRT-PCR. The influence of sub-MIC of coumarin on the relative expression of caerbapenemase genes (*bla*_VIM−2_, *bla*_NDM−1_ and *bla*_OXA−9_). The data represent the mRNA expression of each gene both in presence and absence of coumarin. The expression values were normalized using the 16 S rRNA as a house keeping gene. *** Indicates significance at *P* < 0.001

The qRT-PCR was conducted to evaluate the effect of coumarin on the expression level of carbapenemase-encoding genes in *K. pneumoniae* isolates. Results showed that the expression of tested genes significantly decreased in coumarin treated bacteria as compared to untreated cells. Coumarin (500 µg/mL) decreased the expression of both *bla*_OXA−9_ and *bla*_VIM−2_ by more than 50% with *bla*_*OXA−9*_ being the least expressed gene. But this concentration was not enough to inhibit *bla*_NDM−1_ expression. On the other hand, coumarin at a concentration of 1000 µg/mL significantly reduced the expression of all tested genes with *bla*_OXA−9_ being the least expressed and *bla*_VIM−2_ showing the highest expression level (Fig. 4).

### Molecular docking analysis

**Fig. 5:**
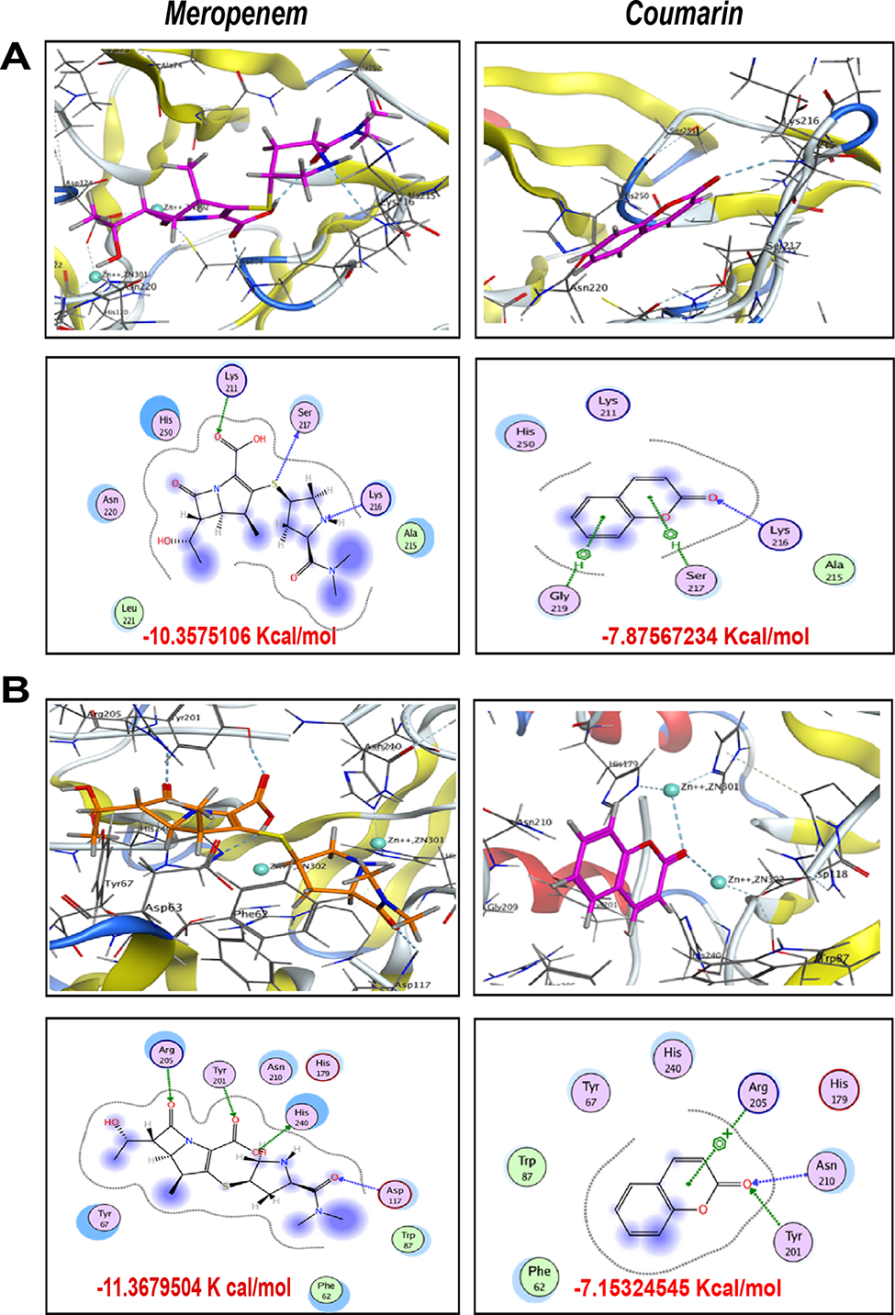
Putative binding modes (3D in upper panel and 2D in the lower panel) and binding free energy of both meropenem and coumarin with class B carbapenemases represented by **(A)** NDM-1, **(B)** VIM-2

The docking study has been conducted to inspect the binding mode of each ligand with its corresponding macromolecule. Meropenem ((4 S,5R,6R)-3-(((3R,5R)-5-(dimethylcarbamoyl)pyrrolidin-3-yl)thio)-6-((S)-1-hydroxyethyl)-4-methyl-7-oxo-1-azabicyclo [3.2.0]hept-2-ene-2-carboxylic acid) as an organosulfur compound, was docked onto the active site of the crystal structure of NDM-1 (PDB:3SPU), and the results (Fig. 5A) revealed that the Lewis bases, *sp*^*3*^-hybridized nitrogen atom of the pyrrolidine ring and *sp*^*2*^-hybridized oxygen atom of the carboxylic group have constructed two H-bonds with the backbone of the conserved amino acid Lys216 and the side chain of the conserved amino acid Lys211, respectively. Besides, the sulfur bridge showed a non-covalent sulfur-σ-hole-bonding with Ser217 enhancing the total stability of ligand/ receptor complex to score free binding energy − 10.3575 Kcal/mol. On the other hand, docking of Coumarin (2*H*-chromen-2-one) as a cyclic ester compound demonstrated one H-bond between the *sp*^*2*^-hybridized oxygen and Lys216, and two arene-H bonds between the heterocycle pyran ring and benzene ring from the ligand, and the side chains of Ser217 and Gly219 from the receptor side respectively ending up with free binding of energy − 7.8757 Kcal/mol.

However, docking results of Meropenem against the crystal structure of VIM-2 in complex with (2R)-1-(2-Benzyl-3-mercaptopropanoyl) piperidine-2-carboxylic acid (PDB: 5O7N) exhibited four H-bonds between *sp*^*2*^-hybridized oxygen of the carbamoyl moiety, hydrogen atom of the carboxylic group, *sp*^*2*^-hybridized oxygen of the carboxylic group and *sp*^*2*^-hybridized oxygen of the fused heterocycle azetidin-2-one from the ligand side and Asp117, His240, Tyr201 and Arg205 respectively from the receptor side giving rise to achieve free binding energy − 11.3679 Kcal/mol. While Coumarin showed bifurcated H-bond between *sp*^*2*^-hybridized oxygen and Asn210 and Tyr201 in addition to arene-cation bond between the heterocycle pyran ring and Arg205 that improved the stability of ligand/ receptor complex to score − 7.1532 Kcal/mol (Fig. 5B).

**Fig. 6:**
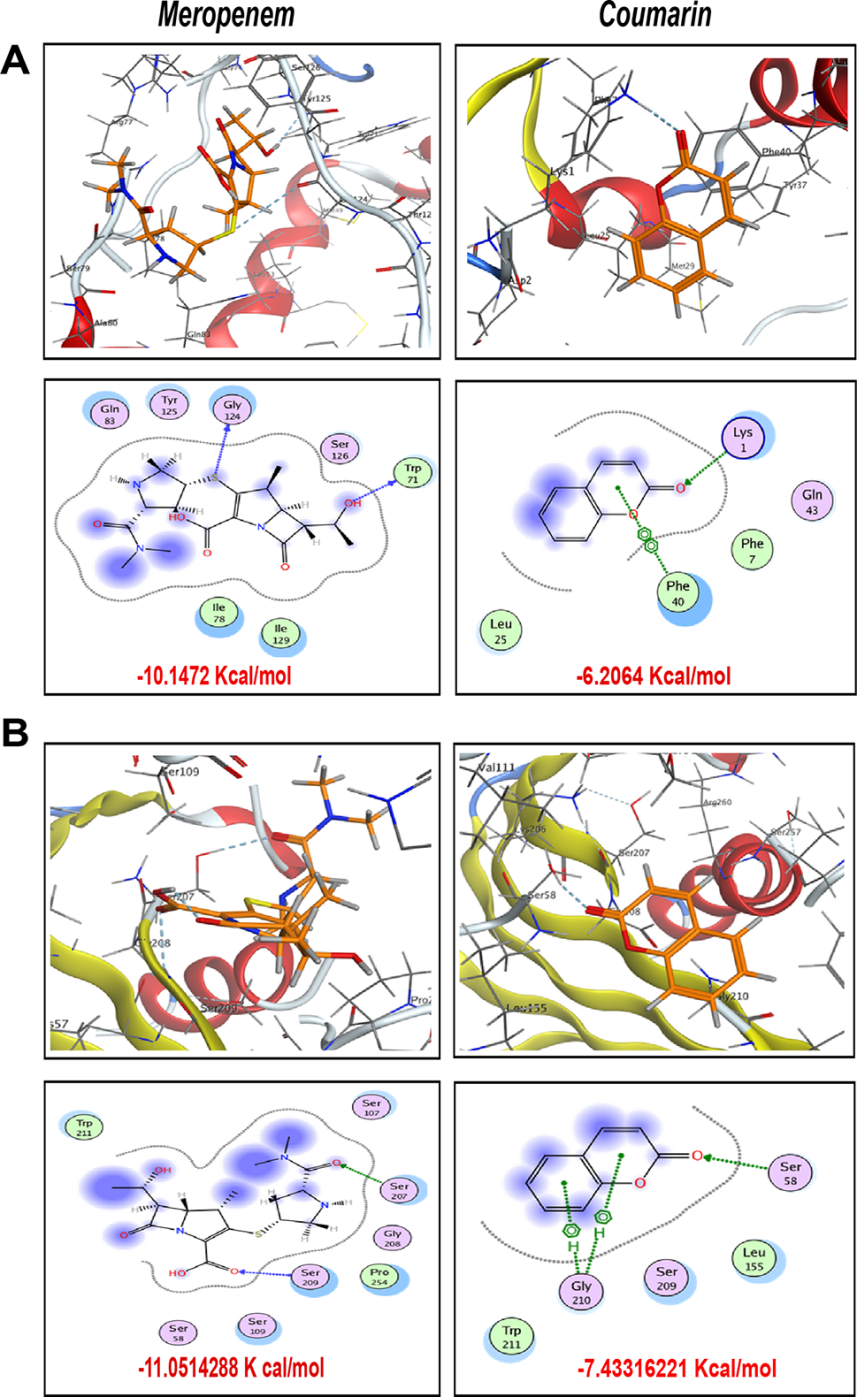
Putative binding modes (3D in upper panel and 2D in the lower panel) and binding free energy of meropenem and coumarin with class D carbapenemases represented by **(A)** OXA-48, **(B)** OXA-9

Yet, docking results (Fig. 6A) of Meropenem onto the predicted protein class D OXA-48 carbapenemase (A0A809EUY3 · A0A809EUY3_KLEPN) revealed one H-bond between the hydrogen atom of the hydroxyl group in the hydroxyethyl moiety and the backbone of the conserved amino acid Trp71 as well as noncovalent sulfur-σ-hole-bonding between the sulfur bridge and the backbone of Gly14 that enhanced the fitting of the ligand inside the core of the active site and gave free binding energy − 10.1472 K cal/mol. While Coumarin displayed one H-bond between *sp*^*2*^-hybridized oxygen and Lys1, as well as an arene-arene bond between pyran ring and the aromatic conserved amino acid Phe40, ending up with binding energy − 6.2064 Kcal/mol.

On the other hand, as shown in (Fig. 6B), docking results of Meropenem against the 3D structure of blaOXA-9 of *K. pneumoniae* (M9V370 · M9V370_KLEPN) inferred from homology, revealed two conspicuous H-bonds; first between *sp*^*2*^-hybridized oxygen of the carbamoyl moiety and the H-bond donor side chain of the conserved amino acid Ser207, and the latter between *sp*^*2*^-hybridized oxygen of the carboxylic group and the conserved amino acid Ser209. Additionally, the hydrophobic/ hydrophilic interactions revealed from the blue-shaded moieties from ligand side and the cyan-shaded moieties from receptor side improved the overall recognition of the ligand inside the active site to achieve free binding energy − 11.0514 Kcal/mol. While Coumarin demonstrated one H-bond between *sp*^*2*^-hybridized oxygen and the conserved amino acid Ser58 along with a bifurcated H-arene bond constructed between Gly210 and the two fused rings of the ligand paving the way to score − 7.43316221 Kcal/mol.

## Discussion

Antimicrobial resistance (AMR) is a major healthcare problem worldwide, threatening more and more lives every year, in addition to billions of dollars spent to limit the extent of this crisis [49]. The overuse of antibiotics during COVID-19 pandemic particularly in low- and middle-income countries raised devastating effects on AMR management [50]. For instance, the incidence of CRKP was significantly increased during the COVID-19 period by 4.8 times [51]. CRKP is one of the urgent threats causing a high rate of mortality, and presenting a major threat to public health [52, 53]. This study aimed to restore the activity of meropenem as one of the last resort antibiotics by finding a potential inhibitor of carbapenemases; the most common causes of carbapenem resistance [54]. Phenolic compounds of natural source were found to have a promising antibacterial activity. For example, tannic acid, epigallocatechin gallate, quercetin, and epicatechin showed a significant inhibitory effect on β-lactamases both in vitro and *in silico* analysis [55]. Coumarin as one of the phenolic compounds, was tested in this study for its potential carbapenemase inhibitory activity.

*K. pneumoniae* isolates included in the current study were MDR, in addition to being meropenem resistant. *K. pneumoniae* strains isolated herein showed a worrisome level of meropenem resistance with higher MICs (64 to 512 µg/mL) with isolate 4 K exhibited the highest MIC (512 µg/mL). These results were consistent with another study reported that carbapenem resistance in Egypt is on rise reaching crisis level with 93.4% of CRK [56]. Of note that, the MIC values reported herein were higher than those reported previously by Gandor and his coworkers who reported that meropenem MICs ranged from 0.002 to 32 µg/mL [57]. These CRK isolates were further investigated for presence of three carbapenemases encoding genes (*bla*_NDM−1_, *bla*_VIM_, and *bla*_OXA_) with the highest prevalence in Egypt as reported with various epidemiological studies. According to a recent study conducted in Egypt [16], *bla*_OXA_ was the most common (15.5%), followed by *bla*_VIM_ (15%), *bla*_IMP_ (7.5%), *bla*_KPC_ (4%), and *bla*_NDM_ (3.8%). In addition, Raheel and coworkers reported that the *bla*_OXA_ was the gene with the highest frequency (96.2%), while the *bla*_KPC_ gene (7.5%) was the lowest [15].

While the MIC of the coumarin was higher for all tested CRK isolates (≥ 8192 µg/mL), sub-MIC of coumarin (500–1000 µg/mL) could efficiently inhibit meropenem hydrolytic activities. Coumarin inhibited carbapenemases when co-incubated with the pooled periplasmic extract of bacterial cells and showed a mean inhibitory percentage of 57.33% ± 7.59, with isolate 5 K having the highest inhibition percentage. These findings suggest that coumarin at concentration of 500–1000 µg/mL can inhibit carbapenemases through direct binding to the target enzymes as suggested by the docking analysis. It is important to know that the tolerable dose of coumarins is 0.1 mg/kg of body weight based on the toxicological properties of coumarins in humans [58]. In addition, previous research groups have made several structural modifications of coumarin nucleus to develop more effective coumarins with lower MICs and diminished toxicity [30].

In addition, culturing of tested *K. pneumoniae* isolates in presence of sub-MICs of coumarin significantly reduced meropenem hydrolysis which further suggests that coumarin could have an inhibitory action on the expression of carbapenemases genes. This explanation was supported by qRT-PCR that was performed to investigate the effect of coumarin on the expression of carbapenemases genes. Coumarin at a concentration of 1000 µg/mL showed a higher inhibitory effect on all tested genes (*bla*_OXA−9_, *bla*_NDM−1_ and *bla*_VIM−2_). Moreover, 500 µg/mL of coumarin was enough to cause a significant reduction in the expression of both *bla*_OXA−9_ and *bla*_VIM−2_.

The synergism between coumarin and meropenem against *K. pneumoniae* was further characterized by the combined disk method. The inhibition zone around the meropenem disk was significantly increased in a dose dependent manner when CRK was cultured in presence of coumarin relative that of meropenem alone. This synergism was further manifested using the checkerboard MIC assay. The FIC index for coumarin (500 µg/mL)-meropenem combination was < 0.5, indicating synergism except for three isolates (1 K, 2 K, and 4 K) which carry *bla*_NDM−1_ that showed non-significant reduction on mRNA expression at this concentration (500 µg/mL). On contrary, combination of coumarin at a concentration of 1000 µg/mL with meropenem showed FICI values < 0.5 in all isolates, indicating synergism. Importantly, coumarin at a concentration of 1000 µg/mL rendered almost all tested *K. pneumoniae* isolates sensitive to meropenem (with MIC < 4 µg/mL) according to CLSI standards [31] except for isolate 4 K which could encode another resistance mechanism to carbapenem such as efflux pump [19, 59, 60]. Similarly, a recent study showed that coumarin derivatives could potentiate the antibacterial activity of norfloxacin against MDR *Staphylococcus aureus* isolates [61]. In addition, quercetin which is structurally related to coumarin and is known to have a carbapenemase inhibitory activity in Gram negative bacteria [19, 59]. and demonstrated a synergistic interaction with colistin, amikacin and meropenem against resistant *Acinetobacter baumannii* [62].

In support of previous findings, *in silico* analysis of coumarin showed low binding energies to tested carbapenemases as shown by molecular docking. NDM1, VIM-2, OXA-48 and OXA-9 showed a free binding energy of -7.8757, -7.1532, -6.2064 and − 7.4331 Kcal/mol, respectively. Of note, all the tested carbapenemases have a high tendency to hydrolyze Meropenem with a free binding energy of more than − 10 Kcal/mol, for all carbapenemases. However, Coumarin as a small molecule showed competitive inhibition for these enzymes with its characteristic binding profile. Hence, if Coumarin co-administered with Meropenem it would improve the efficacy of Meropenem, as it will spare it from detrimental effect of these hydrolyzing enzymes.

All the structural compartments of coumarin were found to be advantageous moieties and shared in stabilization of ligand/receptor complex. The cyclic ester was found to be the maestro moiety in coumarin as it persistently shared in fixation of ligand inside the spot of the receptor through H-bonding. Similarly, a molecular docking study on the phenolic compound mangiferin showed that mangiferin interacted with NDM-1 catalytic amino acid residues through hydrogen bonding and hydrophobic interactions. Mangiferin was found to have a good docking score (-9.12 Kcal/mol) with NDM-1, compared to a docking score of -8.77 Kcal/mol for meropenem, indicating the possible biological activity of mangiferin as a carbapenemase inhibitor [63].

In conclusion, this study showed that coumarin could help to render CRK sensitive to meropenem as suggested by its inhibitory activity on both the hydrolytic activity and the expression of carbapenemases making it a possible solution to overcome carbapenems resistance.

### Electronic supplementary material

Below is the link to the electronic supplementary material.

Supplementary Material 1

## Data Availability

The datasets used /or analyzed in the current study are available from the corresponding author on reasonable request. The 16 S rRNA sequences of the strains were submitted to GenBank https://www.ncbi.nlm.nih.gov/, and they are publicly available under accession numbers: ON798797 (1 K) https://www.ncbi.nlm.nih.gov/nuccore/ON798797, ON798798 (2 K) https://www.ncbi.nlm.nih.gov/nuccore/ON798798, ON798799 (3 K) https://www.ncbi.nlm.nih.gov/nuccore/ON798799, ON798800 (4 K) https://www.ncbi.nlm.nih.gov/nuccore/ON798800, ON798801 (5 K) https://www.ncbi.nlm.nih.gov/nuccore/ON798801, and ON798802 (6 K) https://www.ncbi.nlm.nih.gov/nuccore/ON798802.
