# Supplementary material for: Phenotypic, molecular, and in silico characterization of coumarin as carbapenemase inhibitor to fight carbapenem-resistant Klebsiella pneumoniae
Source: BMC Microbiol. 2024 Feb 27;24:67. doi: 10.1186/s12866-024-03214-7 (PMC10898048; doi:10.1186/s12866-024-03214-7)
Supplement: Supplementary file 1 — Supplementary Material 1 [file 12866_2024_3214_MOESM1_ESM.docx]

**Phenotypic*,* molecular, and *in silico* characterization of coumarin as carbapenemase inhibitor to fight carbapenem-resistant *Klebsiella pneumoniae***

## **Mahmoud Saad Abdel-Halim ^1, *^, Amira M. El-Ganiny ^1^ , Basem Mansour ^2^, Galal Yahya ^1^, Hemat K. Abd El Latif ^1^, Momen Askoura ^1^**

^1^ Microbiology and Immunology Department, Faculty of Pharmacy, Zagazig University, 44519, Zagazig, Egypt.

**^2^** Pharmaceutical Chemistry Department, Faculty of Pharmacy, Delta University for Science and Technology, Gamasa 11152, Egypt

* Corresponding author e-mail: mahmoudsaad@zu.edu.eg

[mahmoudsaadelmasry11@gmail.com](mailto:mahmoudsaadelmasry11@gmail.com)

Institutional Phone number: 002-055-2303266.

Mobile: 00201023653456.

ORCID ID: [https://orcid.org/0000-0002-3944-8465](https://orcid.org/0000-0002-2694-1998)


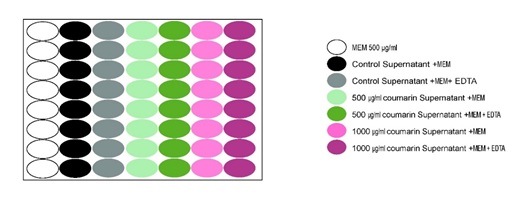


**Supplementary** **Fig. 1: Coumarin enzyme inhibition activity:** schematic diagram of the reaction plate.

**Supplementary Table 1. Antimicrobial susceptibility profile of *K. pneumoniae* isolates.**

| **Isolate’s**  **Code** | **Anti-microbial agents** | | | | | | | | | | | | | | |
| --- | --- | --- | --- | --- | --- | --- | --- | --- | --- | --- | --- | --- | --- | --- | --- |
|  | **MEM** | **CFP** | **CRO** | **TZP** | **FEP** | **ATM** | **OFX** | **LEV** | **AK** | **CN** | **TGC** | **TE** | **SXT** | **AZM** | **C** |
| **1K** | R | R | R | R | R | R | R | R | R | R | R | R | R | R | S |
| **2K** | R | R | R | R | R | R | R | R | S | S | R | R | I | R | I |
| **3K** | R | R | R | R | R | S | R | R | R | S | I | R | R | R | I |
| **4K** | R | R | R | R | R | R | R | R | R | R | R | R | R | R | S |
| **5K** | R | R | R | R | R | R | R | R | R | R | I | I | R | R | R |
| **6K** | R | R | R | R | R | R | R | R | R | R | S | I | R | R | S |

**R= Resistant S= Sensitive I= Intermediate**

meropenem (MEM, 10 μg), ceftriaxone (CRO, 30 μg), piperacillin-tazobactam (TZP, 100/10 μg), cefepime (FEP, 30 μg), cefoperazone (CFP, 75 μg), aztreonam (ATM, 30 μg), gentamicin (GN, 10 μg), amikacin (AK, 30 μg), trimethoprim-sulfamethoxazole (SXT, 1.25/ 23.75 μg), tetracycline (TE, 30 μg), tigecycline (TGC, 15 μg), levofloxacin (LEV, 5 μg), ofloxacin (OFX, 5 μg), and chloramphenicol (C, 30 μg), azithromycin (AZM, 15 μg).
